# Vaccine-Mediated Protection of Mice Against African and Asian Clinical Strains of *Cryptococcus neoformans*

**DOI:** 10.3390/jof11120886

**Published:** 2025-12-16

**Authors:** Diana Carlson, Ruiying Wang, Zachary Hastings, Lorena V. N. Oliveira, Maureen M. Hester, Nicolle Rodriguez, Gabriel Kristian Pedersen, Jennifer L. Tenor, John R. Perfect, Charles A. Specht, Stuart M. Levitz

**Affiliations:** 1 Department of Medicine, University of Massachusetts Chan Medical School, Worcester, MA 01655, USA; diana.carlson@umassmed.edu (D.C.); ruiying.wang@umassmed.edu (R.W.); zachary.hastings@umassmed.edu (Z.H.); lorena.ramalho@umassmed.edu (L.V.N.O.); maureen.hester@umassmed.edu (M.M.H.); nicolle.rodriguez1@outlook.com (N.R.); charles.specht@umassmed.edu (C.A.S.); 2 Center for Vaccine Research, Statens Serum Institut, 2300 Copenhagen, Denmark; gakp@ssi.dk; 3 Department of Medicine, School of Medicine, Duke University, Durham, NC 27708, USA; jennifer.tenor@duke.edu (J.L.T.); john.perfect@duke.edu (J.R.P.)

**Keywords:** fungal vaccines, cryptococcosis, adjuvant, global health, HIV infection, AIDS, meningitis

## Abstract

Infections with strains of the *Cryptococcus neoformans* species complex are responsible for over 100,000 deaths per year, predominantly due to meningitis in immunocompromised individuals. Despite much research, there are no licensed fungal vaccines available. Most experimental cryptococcal vaccine formulations have been tested in preclinical models using laboratory strains of *C. neoformans*, particularly H99 and KN99. However, to be effective, vaccines need to protect against the wide variety of cryptococcal isolates found worldwide, particularly in regions that have the highest burden of infections. Therefore, we explored vaccine-mediated protection of BALB/c mice against experimental cryptococcosis due to six *C. neoformans* strains originally isolated from patients with cryptococcal meningitis in Vietnam, Uganda, and Botswana. Two vaccines were tested: a live-attenuated *C. neoformans* vaccine lacking three chitin deacetylase genes, and a quadrivalent subunit protein vaccine adjuvanted with Cationic Adjuvant Formulation 01. When compared to unvaccinated mice, both vaccines provided significant protection against all six clinical strains. However, the degree of protection varied as a function of vaccine formulation and clinical strain. Lung leukocytes from vaccinated and infected mice had significantly increased antigen-stimulated interferon-gamma production compared with infected but unvaccinated mice. Thus, although the degree of protection varied, two cryptococcal vaccines significantly protected mice against experimental infection with cryptococcal strains representative of regions of the world that account for the majority of cryptococcal meningitis cases found globally. These data provide preclinical support for trialing vaccines in persons at high risk for developing cryptococcosis.

## 1. Introduction

Virtually all cases of cryptococcosis are caused by *Cryptococcus neoformans* and the closely related species complex *C. gattii* [1]. The opportunistic pathogen predominantly infects hosts with CD4^+^ T cell dysfunction. Persons with AIDS are particularly vulnerable. It is estimated that in 2020, 112,000 people with HIV died of cryptococcal meningitis, accounting for 19% of AIDS-related deaths [2]. Initial exposure occurs following the inhalation of yeast cells or basidiospores from environmental sources such as trees and soil contaminated with pigeon guano [1]. Two varieties of *C. neoformans*, var. *grubii* (serotype A) and var. *neoformans* (serotype D), have been recognized with >95% of human cases caused by var. *grubii*; however, some taxonomists posit that the two varieties are distinct species [3,4,5]. Three genetically distinct subpopulations of *C. neoformans* var. *grubii*, designated VNI, VNII, and VNB, have been identified [6,7]. The VNI and VNII lineages are found worldwide, while VNB is mostly found in sub-Saharan Africa, particularly Botswana [5,6,7,8].

Despite the need, there are currently no licensed fungal vaccines [9]. In preclinical studies, several laboratories, including ours, have developed vaccines which protect mice in experimental models of cryptococcosis. We have studied both attenuated whole organism vaccines and subunit vaccines. For the former, an attenuated *C. neoformans* var. *grubii* strain KN99 was constructed in which three chitin deacetylase (Cda) genes (*Cda1*, *Cda2*, and *Cda3*) were deleted. The resulting avirulent strain, *cda1*Δ*2*Δ*3*Δ (heretofore referred to as “cda123”), lacks cell wall chitosan and is cleared from the lungs of severely immunodeficient mouse lines [10,11]. Pulmonary vaccination with cda123 protects several strains of mice against an otherwise lethal challenge with KN99 [10,11]. The subunit vaccines consist of cryptococcal proteins recombinantly expressed in *E. coli*, adjuvanted with Cationic Adjuvant Formulation 01 (CAF01). We have shown that subcutaneous vaccination with four recombinant antigens adjuvanted with CAF01 stimulates robust, antigen-specific CD4^+^ T cell immune responses and protects mice against KN99 challenge [12,13,14]. The four-antigen vaccine, hereby referred to as “4-Ag”, contains Cda1 and Cda2, carboxypeptidase 1 trimmed of its region of human homology (Cpd1Δ), and Barwin-like domain protein 4 (Blp4). CAF01 is a liposome-based Mincle agonist adjuvant composed of the cationic quaternary ammonium salt N,N’-dimethyl-N,N’-dioctadecylammonium and the glycolipid mycobacterial immunomodulator α,α′-trehalose 6,6′-dibehenate [12,15].

KN99 is a genetically defined virulent laboratory strain derived from a clinical strain, H99, that was originally isolated in the United States from a patient with lymphoma [16,17]. It is important to test vaccine candidates using diverse clinical strains isolated from regions of the world where disease is most prevalent. Clinical and environmental isolates of *C. neoformans* differ in their virulence in mouse models [18,19,20]. Additionally, there is evidence from human studies that the fungal strain genotype informs clinical outcomes [21]. Therefore, in the present studies, we assembled a panel of six virulent *C. neoformans* strains isolated from patients with cryptococcal meningitis in Vietnam, Uganda, and Botswana, analyzing two strains from each country. We then tested the ability of our two cryptococcal candidate vaccines to protect mice against pulmonary challenge with the six strains. We found that both vaccines significantly protected mice, as measured by prolonged survival and decreased fungal organ burdens. However, the degree of protection differed as a function of cryptococcal strain and vaccine. Moreover, lung leukocytes isolated from vaccinated and infected mice had robust antigen-specific interferon-gamma (IFNγ) responses following ex vivo stimulation.

## 2. Materials and Methods

*C. neoformans* strains. Strains used to infect mice are listed in Table 1. Strains BK80 and BMD1338 were from Jeremy Day (University of Oxford) [20,22]. Strains UgCl302, UgCl395 [19,23], and the reference strain, KN99 [24], were obtained from Kirsten Nielsen (Virginia Tech University). Strains PMH1063 and PMH1065 were from Jennifer Tenor and John Perfect (Duke University) and their collection of clinical strains from Princess Marina Hospital in Gaborone, Botswana [25]. Strain BMD1338 was isolated from a patient without known immunodeficiency [20]. The other clinical strains were isolated from persons with HIV coinfection. Stock cultures of the strains were stored at −80 °C in 25% glycerol.

Mouse Vaccination and Infection Model. BALB/c mice were purchased from the Jackson Laboratory (Bar Harbor, ME) and were housed and bred in the animal facilities of the University of Massachusetts Chan Medical School (UMCMS) in a specific pathogen-free environment. Animal husbandry and experimental procedures were conducted according to the protocols approved by the Institutional Animal Care and Use Committee at UMCMS. Male and female mice were used in approximately equal numbers in all the experiments. At 6–10 weeks of age, mice were vaccinated with either live-attenuated cda123 or a combination of four recombinant proteins with Tris Buffer (10 mM, pH 7.0, supplemented with 2% glycerol) and CAF01 adjuvant (Statens Serum Institute, Copenhagen, Denmark). The live-attenuated cda123 was prepared and administered as a single orotracheal dose containing 2 × 10^7^ in 50 μL PBS as described [10]. The 4-Ag vaccine was prepared and administered as in previous studies [12]. Briefly, His-tagged antigens were recombinantly expressed in *E. coli* and purified on a nickel column. The 4-Ag vaccine consisted of two separate injections adjuvanted with CAF01. One injection contained a combination of Cda1 and Cda2, while the other contained a combination of Cpd1Δ and Blp4. The 4-Ag vaccine contained 5 μg of each protein. Injections were administered subcutaneously (100 μL each) and were repeated at two-week intervals for a total of three vaccinations. Unvaccinated mice served as controls.

To prepare the inoculum to infect the mice, *C. neoformans* strains were grown in yeast-peptone-dextrose (YPD) broth in a shaker for 18 h at 30 °C, washed in PBS, counted, and resuspended in PBS containing 4 × 10^5^ yeast cells/mL. The mice were then challenged orotracheally with 50 µL of the suspension (containing 2 × 10^4^ yeast cells). Mice were monitored daily. Euthanasia with CO_2_ asphyxiation was performed for mice that reached humane endpoints and time-point organ collection experiments.

Assessment of Organ Fungal Burden and Antigen-stimulated IFNγ Production. Mice were euthanized, exsanguinated by cardiac puncture, and spleens, lungs, and brains were harvested. Splenocytes and lung cells were prepared as described [12,26]. Briefly, spleens were gently macerated manually through a 70 µm strainer. Lung cells were prepared using the MACS Lung Dissociation Kit for mice, according to the manufacturer’s instructions (Miltenyi Biotec, Bergisch Gladbach, Germany). The remaining lung cells were passed through a 70 µm cell strainer. Then, 200 µL portions of the lung and spleen samples were set aside to assess fungal burden. Leukocytes were then enriched on a 67% and 40% density gradient (Percoll, Cytiva, Marlbourough, MA, USA) by centrifugation at 800× *g* for 20 min without braking, followed by the collection of cells from the interphase. Single cell lung leukocytes were washed and resuspended in complete medium (RPMI 1640 supplemented with 10% FBS, 1% GlutaMAX, 1% HEPES, and 1% Penicillin–Streptomycin, all purchased from ThermoFisher Scientific, Waltham, MA, USA). Cell concentration and viability were then determined by Trypan Blue stain and a TC20 cell counter (Bio-Rad, Hercules, CA, USA). Brain samples were collected and homogenized in 2 mL of PBS supplemented with 2% Penicillin–Streptomycin. Fungal burdens in spleens, lungs, and brains were determined by counting CFUs following dilution and spreading on Sabouraud dextrose agar plates. The lower limit of detection was 20 CFUs for lungs and 10 CFUs for brains and spleens. If no CFUs were detected, a value for the lower limit of detection was assigned.

Heat-killed *C. neoformans* strains for use as ex vivo stimuli were prepared as described [26,27]. Each strain was cultured in a shaker for 18 h in YPD at 30 °C. A 20 μL aliquot was then taken and put into 4 mL of fresh broth and cultured for an additional 48 h. Cells were counted and diluted with PBS to 2.6 × 10^7^ cells/mL, which corresponds to a dry weight of 1 mg/mL. The strains were then heat-killed at 70 °C for 30 minutes. Fungal death was confirmed by plating samples on Sabouraud dextrose agar and demonstrating the absence of colony forming units (CFU). Aliquots were stored at −80 °C until used for assays.

Antigen-stimulated IFNγ production by lung leukocytes was determined as in our previous studies [12,26]. Cells were cultured in round-bottomed 96-well plates and stimulated with the indicated antigens in a final volume per well of 200 µL RPMI complete medium supplemented with 0.5 µg/mL amphotericin B (ThermoFisher, Waltham, MA, USA). Final concentrations of recombinant proteins and heat-killed strains were 5 µg/mL and 50 µg/mL, respectively. Lung leukocytes were cultured for 18 h at 4 × 10^5^ cells/well. The cultures were maintained at 37 °C within a controlled humidified atmosphere with 5% CO_2_. Supernatants were then collected for IFNγ analysis via ELISA (Mouse DuoSet ELISA Kits, R&D Systems, Bio-Techne, Minneapolis, MN, USA).

Graphs and Statistics. GraphPad Prism version 10.1.1 (GraphPad Software Inc., Dotmatics, San Diego, CA, USA) was used to create survival curves and graphs and to perform statistical analysis. Kaplan–Meier survival curves were analyzed using the Mantel–Cox log-rank test, while the organ fungal burdens were analyzed using the Kruskal–Wallis test with multiple comparisons. For all statistical analyses, a *p* value of <0.05 following corrections for multiple comparisons was considered statistically significant.

## 3. Results

**Figure 1:**
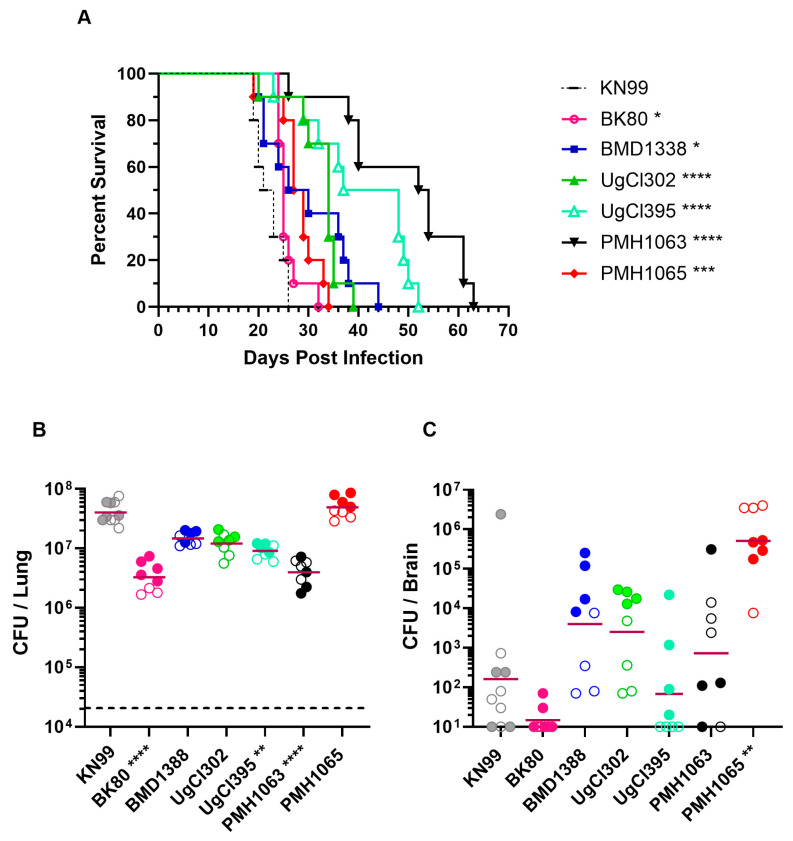
Survival and organ fungal burdens following pulmonary challenge with clinical *C. neoformans* strains. BALB/c mice were inoculated with 2 × 10^4^ CFUs orotracheally with the strains shown in Table 1. (**A**) Mice were monitored over a period of 70 days post infection (DPI) for survival assessment. (**B**,**C**) Mice were euthanized at 14 days post infection to assess fungal burdens in the lungs and brain, respectively. The horizontal dashed line in (**B**) denotes the fungal inoculum. The horizontal bar represents geometric means. Data are the results of independent duplicate experiments, with 5 mice/group for the survival experiments and 3–5 mice/group for the CFU experiments. For (**B**,**C**), the first experiment is shown with filled circles while the second experiment is in open circles. For (**B**), the dashed horizontal line denotes the inoculum used to infect the mice. Statistical comparisons are in relation to the KN99 control strain. In (**A**), Kaplan–Meier curves were compared using the Mantel–Cox log-rank test. For (**B**,**C**), the Kruskal–Wallis test was used to determine significance. Only comparisons that are significant are noted (**** *p* < 0.0001, *** *p* < 0.001, ** *p* < 0.01, * *p* < 0.05).

Two strains each from Vietnam, Uganda, and Botswana were chosen for study, along with the reference strain KN99 (Table 1). Initial experiments were designed to demonstrate the virulence of the isolates in unvaccinated BALB/c mice following orotracheal challenge with 2 × 10^4^ yeast cells. While all mice succumbed by day 63 post-infection, there was variability in median survival, with the mice infected with clinical strains surviving significantly longer than those infected with KN99 (Figure 1). Based on times to 100% mortality, strains BK80 and PMH1065 were the most virulent, followed by UgCl302, BMD1338, UgCl395 and PMH1063. Next, we examined lung and brain CFUs at 14 days post infection. For each of the strains, the mice had lung CFU at least 100 times higher than the inoculum, with mice infected with BK80, UgCl395, and PMH1063 having significantly fewer lung CFUs than KN99-infected mice. PMH1065-infected mice had significantly higher CFU in the brain than KN99-infected mice. Brain CFUs from mice infected with BMD1388, UgCl302, and PMH1063 trended higher, but this difference was not statistically significant.

**Figure 2:**
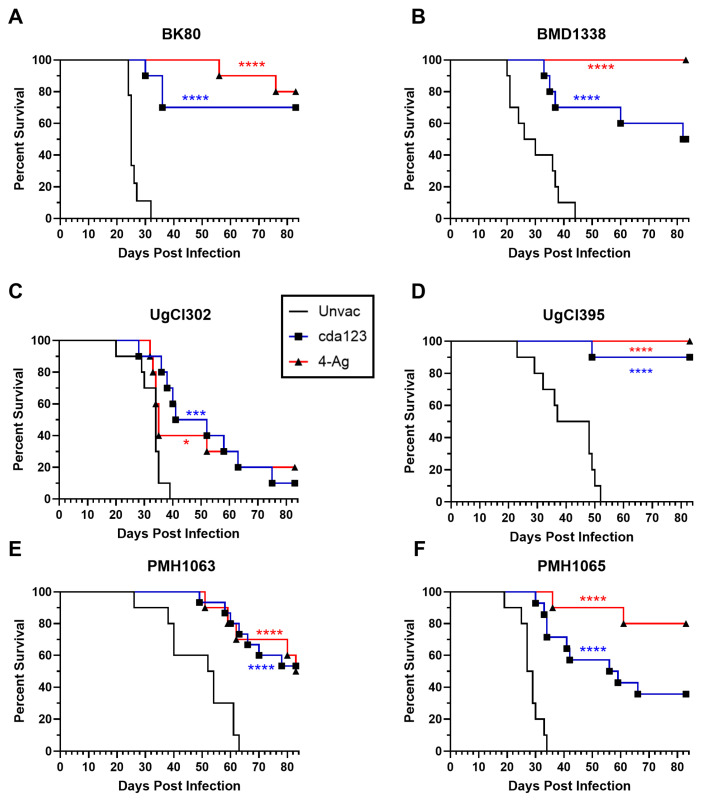
Effect of vaccination on survival following challenge with cryptococcal clinical strains. Mice received either the live-attenuated cda123 vaccine or the CAF01-adjuvanted Cda1/Cda2 and Cpd1Δ/Blp4 vaccine (4-Ag) as described in the Methods section. Mice were challenged orotracheally 6 weeks after the cda123 vaccination or 2 weeks after the last dose of 4-Ag vaccination with 2 × 10^4^ cells of the indicated *C. neoformans* strain. Mice were then monitored for 83 days post infection (DPI) for survival. All vaccinated groups exhibited statistically significant survival compared to their unvaccinated counterparts. For reference, survival curves for unvaccinated (Unvac) mice (shown in Figure 1) are depicted as solid black lines. (**A**) BK80. (**B**) BMD1338. (**C**) UgCl302. (**D**) UgCl395. (**E**) PMH1063. (**F**) PMH1065. Data represent two (for (**A**–**D**)) or three (for (**E**,**F**)) independent experiments, with four to five mice in each experimental group. Kaplan–Meier curves were compared using the Mantel–Cox log-rank test. Asterisks denote statistical significance comparing vaccinated and unvaccinated groups (**** *p* < 0.0001, *** *p* < 0.001, * *p* < 0.05). Results parsed for the sex of the mice can be found in Supplementary Table S1.

We next determined whether our two candidate cryptococcal vaccines, the live-attenuated cda123 vaccine and the 4-Ag CAF01-adjuvanted vaccine, could protect BALB/c mice challenged with the clinical cryptococcal strains, as we reported previously using the reference strain KN99 [12]. Regardless of the infecting clinical strain, vaccination significantly improved survival for all groups (Figure 2). However, the degree of protection varied as a function of vaccine and clinical strain. For example, mice vaccinated with the 4-Ag vaccine had 100% survival following infection with BMD1338 and UgCl395. Conversely, for strain UgCl302, which is closely related to UgCl395 [19], only 20% of the mice were alive when the experiment was terminated on day 83 post-infection. For mice that received the cda123 vaccine, survival at day 83 post infection ranged from 10% for strain UgCl302 to 90% for strain UgCl395.

At the time of euthanasia, lung and brain CFUs were determined for the mice that survived the infection (Supplemental Figure S1). The best control of infection, as determined by organ CFUs, was seen for mice that received the 4-Ag vaccine and were infected with BK80. For that group, CFUs in the lung were below the infecting inoculum and all but one mouse had undetectable brain CFUs. Other groups had higher CFUs, although there was a lot of intragroup variation.

**Figure 3:**
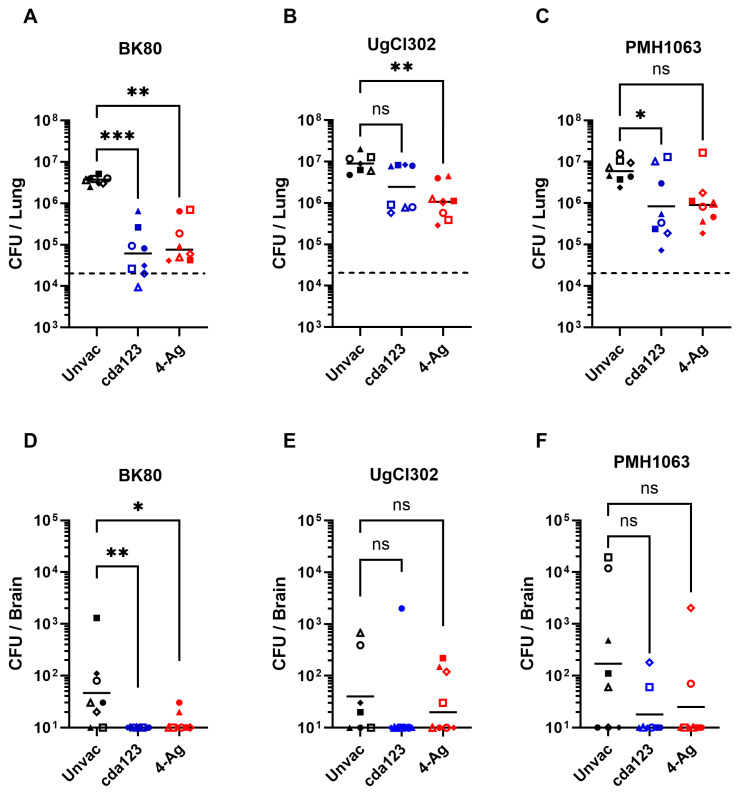
Lung and brain fungal burdens following vaccination and infection with the clinical strains. As in Figure 2, BALB/c mice were vaccinated with either cda123 or 4-Ag and then infected with BK80 (**A**,**D**), UgCl302 (**B**,**E**) and PMH1063 (**C**,**F**). Unvaccinated (Unvac) and infected mice served as controls. Mice were euthanized at 14 DPI to determine the fungal burden in the lungs and brain. Two independent experiments were performed, each with 3–4 mice per group, with each symbol corresponding to an individual mouse. Filled and unfilled symbols represent the separate experiments. Horizontal solid black bars are the geometric means. The horizontal dashed lines in (**A**–**C**) denote the pulmonary inoculum. A non-parametric Kruskal–Wallis test was used to determine the significance of CFUs from vaccinated compared to the unvaccinated groups. Asterisks refer to statistical difference (*** *p* < 0.001, ** *p* < 0.01, * *p* < 0.05, ns, not significant). Results parsed for the sex of the mice can be found in Supplementary Table S2.

Having demonstrated that vaccination improved survival following challenge with the clinical strains, the next set of experiments explored how well the vaccines reduced fungal burden. CFUs in the lungs, spleens, and brains were determined in unvaccinated and vaccinated mice 14 days following infection (Figure 3). For these experiments, we chose one strain each from Vietnam (BK80), Uganda (UgCl302), and Botswana (PMH1063). Fungal burdens in the lungs and brains were decreased in vaccinated mice compared to unvaccinated mice, regardless of infecting strain. However, statistical significance was achieved for only some comparisons. Of the three fungal strains tested, the most robust vaccine-mediated reductions in organ fungal burdens occurred in mice infected with strain BK80. These vaccinated mice also had the closest lung CFUs to the initial inoculum. Survival following vaccination was also greatest in mice that received BK80 compared with UgCl302 and PMH1063 (Figure 2). Not shown in Figure 3: CFUs were undetectable in the spleens of all unvaccinated and vaccinated mice.

**Figure 4:**
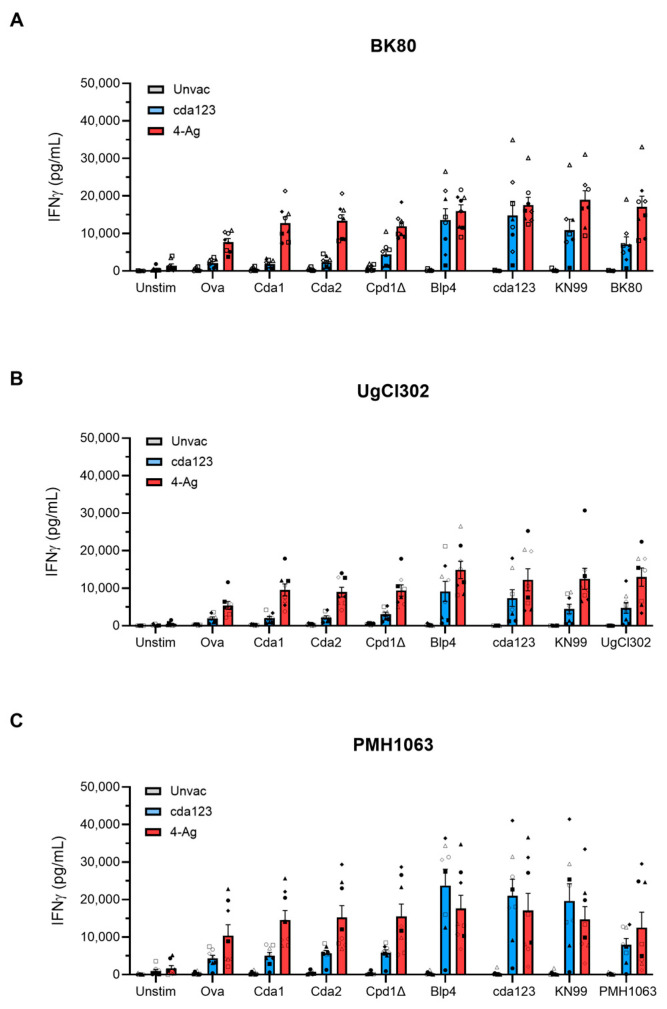
Lung leukocyte IFNγ production after antigen stimulation. Mice were vaccinated and infected with BK80 (**A**), UgCl302 (**B**), and PMH1063 (**C**) as in Figure 3. Control mice were infected but left unvaccinated (Unvac). Leukocytes were prepared from harvested lungs on 14 days post infection and cultured in complete media with amphotericin B for 18 h. The cells were stimulated with the indicated cryptococcal proteins or heat-killed cryptococcal strains. Controls included unstimulated (Unstim) cells and cells stimulated with recombinant ovalbumin (Ova) prepared in the same manner as the recombinant proteins in the 4-Ag vaccine. IFNy concentrations of culture supernatants were measured by ELISA. Two independent experiments were performed, each with 3–4 mice per group. Bars are means ± SEM. The symbols correlate with the symbols from Figure 3. Results parsed for the sex of the mice can be found in Supplementary Table S2.

IFNγ is required for the protection of cda123 and 4-Ag vaccinated mice against challenge with the KN99 strain [10,12]. Therefore, we measured antigen-stimulated IFNγ release by lung leukocytes (Figure 4). Mice were left unvaccinated or vaccinated with cda123 or 4-Ag. The animals were then given a pulmonary challenge with BK80, UgCl302, or PMH1063. Lung leukocytes were harvested at 14 DPI and simulated with recombinant antigens or heat-killed *C. neoformans* cells (including cda123, KN99, and the infecting strain). His-tagged recombinant ovalbumin (Ova), prepared in the same manner as the recombinant *E. coli* proteins, was used as a control stimulus.

Regardless of the antigen tested, IFNγ release was generally low following stimulation of lung leukocytes from unvaccinated mice infected with BK80, UgCl302, or PMH1063 (Figure 4), which has been seen previously with KN99 infection [12]. In addition, irrespective of the infecting cryptococcal strain, some IFNγ release was seen in Ova-stimulated lung leukocytes from vaccinated mice, suggesting responses to the His-tag or contaminating *E. coli* proteins in the preparation. For mice vaccinated with cda123 and infected with any of the three strains, levels of IFNγ following stimulation of lung leukocytes with recombinant Cda1, Cda2, and Cpd1Δ were similar to those seen following Ova stimulation. However, recombinant Blp4 and the heat-killed cryptococcal strains stimulated significant IFNγ release, suggesting antigen-specific responses to Blp4 developed. For the 4-Ag vaccinated and infected mice, significant IFNγ release was assayed following stimulation with each of the recombinant cryptococcal antigens and heat-killed strains. For each antigen and vaccination group, lung leukocyte IFNγ release was similar when comparing lung leukocytes from mice infected with BK80, UgCl302, and PMH1063.

## 4. Discussion

Most AIDS-related cryptococcosis cases occur in sub-Saharan Africa and Asia and are caused by *C. neoformans* var. *grubii* [2,5,28]. Using phylogenetic analysis, four lineages of *C. neoformans* var. *grubii* have been described: VNI, VNII, VNBI, and VNBII [29]. The first two subpopulations have a worldwide distribution, whereas the VNBI and VNBII lineages are mainly confined to Southern Africa [5,29]. To assemble a panel of clinical isolates for our vaccine studies, we prioritized genotypically and phenotypically defined clinical isolates obtained from regions with high cryptococcal prevalence. An additional criterion was known virulence in mouse models of experimental cryptococcosis. The Ugandan strains, UgCl302 and UgCl395, are in the VNI sequence-type 93 lineage, and have been shown to be highly virulent following intranasal infection of A/J mice [19]. Sequence-type 93 isolates predominate in clinical samples from Uganda and Malawi but are also common in other parts of the world, such as Brazil [22,30]. The Vietnamese strains BMD1338 and BK80 are of the sequence-type 5 and sequence-type 4 lineages, respectively, which are frequently found in Southeast Asia [22,31]. The Botswana strains, PMH1063 and PMH1065, are of the VNBII and VNI lineages, respectively [25]. They were isolated as part of a study of 34 patients with HIV-associated cryptococcal meningitis; 31 of the 34 fungal strains were *C. neoformans* while the remaining three were *C. gattii*. Using RNAseq, genes encoding for two of our subunit vaccine antigens, Cda1 and Blp4, were among the top 50 expressed genes in fungi obtained from the cerebrospinal fluid [25].

Many clinical strains isolated from persons with HIV-associated cryptococcosis are avirulent or hypovirulent in mouse models of disease [19,20,21]. For our vaccination studies, we selected six clinical strains that were virulent in BALB/c mice, as measured by 100% mortality within 70 days and increased fungal burden in the lungs and brain at day 14. This strain collection could prove useful not only for vaccine studies but also for investigations into immune responses, virulence factors, and antifungal therapy. Future studies are needed with clinical strains representing other lineages within the *C. neoformans* species complex, as well as the genetically diverse *C. gattii* species complex. Moreover, in our vaccine studies, mice were infected via the pulmonary route, as most human infections are thought to originate following the inhalation of airborne organisms. Future studies will need to examine other routes of infection.

Compared with unvaccinated mice, mice that received the cda123 and 4-Ag vaccines were significantly protected after challenge with the six clinical strains. However, the degree of protection afforded by vaccination differed among the strains. Interestingly, the disparity was most evident among the two Ugandan strains, UgCl302 and UgCl395, both of which are of the VNI sequence-type 93 lineage. Survival at the termination of the study was 90–100% in vaccinated mice challenged with UgCl395, but only 10–20% in vaccinated mice challenged with UgCl302. Closely related strains can differ markedly in terms of mouse virulence [7,18,19,20]. Using a closely related collection of *C. neoformans* sequence-type 93 isolates, mouse virulence could be mapped to single nucleotide polymorphisms in specific genes, some of which also correlated with IFNγ levels upon infection [7]. Our findings extend this observation to include vaccine-mediated protection. Taken together, the studies suggest that caution must be applied before positing that a single strain is representative of a cryptococcal species, variety, or lineage [32,33,34].

Lung leukocytes obtained from mice vaccinated and infected with any of the three clinical strains tested produced robust IFNγ when stimulated with vaccine antigens or heat-killed *C. neoformans* strains. This cytokine response is similar to that seen in vaccinated mice infected with *C. neoformans* strain KN99 [10,12,26,27]. In experimental models of cryptococcosis using *C. neoformans* strain KN99, IFNγ is required for protection mediated by the cda123 and 4-Ag vaccines [10,27]. A vaccine consisting of the H99 strain of *C. neoformans* recombinantly engineered to express murine IFNγ is highly protective in murine infection models [35]. There is strong evidence for the importance of IFNγ in human infection cases, as phase 2 clinical trials of adjuvant IFNγ therapy in patients with HIV and cryptococcal meningitis demonstrated salutary effects with regard to fungal clearance [36,37]. Moreover, peripheral blood mononuclear cells (PBMCs) obtained from subjects with a history of cryptococcosis have greater cryptococcal antigen-stimulated IFNγ production compared with PBMCs from healthy controls [26]. Given the importance of IFNγ in clinical settings and preclinical models, our data demonstrating strong antigen-stimulated pulmonary IFNγ release in vaccinated and infected mice provide support for the clinical development of our cryptococcal vaccines.

Our work demonstrates that two leading vaccine candidates protect against cryptococcal strains representative of those found in sub-Saharan Africa and Southeast Asia, regions of the world that account for the majority of cryptococcal meningitis cases found globally. The 4-Ag vaccine performed as well as and, in some cases, better than the cda123 vaccine, as measured by 83-day survival and 14-day fungal burden in the lungs and brain. However, caution needs to be exercised before concluding that one vaccine is superior, as different dosing regimens and vaccine schedules were used. The cda123 vaccine has the advantage of being easy to prepare and only requires a single dose, but has the drawback of needing to be delivered to the lungs using a relatively high inoculum [10,11]. Moreover, live-attenuated vaccines must be used with caution in immunocompromised populations [38]. The 4-Ag vaccine might be easier to advance clinically as it is a traditional subunit adjuvanted protein vaccine [12]. Vaccine formulations using fewer antigens and chimeric antigens are being studied, as having four antigens increases manufacturing expenses. Recently, the protection of mice against challenge with *C. neoformans* strain H99 was demonstrated using an adjuvanted mRNA vaccine encoding for Cda1, one of the antigens in the 4-Ag vaccine [39]. Promising vaccine studies in mice lend support to trialing vaccines in humans at risk for cryptococcosis.

## Figures and Tables

**Table 1:** *C.neoformans* strains used in the study. Country refers to the country in which the strain was originally isolated. ST, sequence-type.

Strain	Country	Lineage
KN99 (Control)	USA	VNIb
BK80	Vietnam	VNIa ST4
BMD1338	Vietnam	VNIa ST5
UgCl302	Uganda	VNIa ST93
UgCl395	Uganda	VNIa ST93
PMH1063	Botswana	VNBII
PMH1065	Botswana	VNI

## Data Availability

The original contributions presented in this study are included in the article/Supplementary Materials. Further inquiries can be directed to the corresponding author.

## Supplementary Materials

The following supporting information can be downloaded at https://www.mdpi.com/article/10.3390/jof11120886/s1, Figure S1. Lung and brain fungal burdens of surviving mice following vaccination and infection. Mice received either the live-attenuated cda123 vaccine or the CAF01-adjuvanted Cda1/Cda2 & Cpd1Δ/Blp4 vaccine (4-Ag) as described in the Methods. Mice were challenged orotracheally 2 weeks after the last vaccination with 2 × 10^4^ cells of the indicated *C. neoformans* strain. Mice were then monitored for 83 days post infection (DPI) for survival. Survival curves are shown in Figure 2. At the termination of the experiment, surviving mice were euthanized and lung (top) and brain (bottom) CFUs determined. The number of survivors ranged from one to ten depending on the infecting strain and the vaccination. Each circle represents organ CFUs in an individual mouse. Bars are geometric means. Unvaccinated mice all died prior to day 83 and are not shown in the figure. The level of detection for lungs and brain was 20 CFUs and 10 CFUs, respectively. The horizontal dashed line in the top graph denotes the pulmonary inoculum.. Table S1. Vaccine Protection as a function of the sex of the mice. The data parse the results shown in Figure 2 according to the sex of the mouse. Results are presented as survivors/total number of mice per group. M, male. F, female. For example, 4/9 F indicates that of 9 vaccinated female mice, 4 were alive at the termination of the study.. Table S2: Sex of the mice used in Figure 3 and Figure 4. M, male. F, female.

## Abbreviations

The following abbreviations are used in this manuscript:

Cda	Chitin deacetylase
cda123	cda1Δ2Δ3Δ avirulent cryptococcus strain
CAF01	Cationic adjuvant formulation 01
4-Ag	Four-antigen vaccine
Cpd1Δ	Carboxypeptidase 1 trimmed of its region of human homology
Blp4	Barwin-like domain protein 4
YPD	Yeast-peptone-dextrose
Ova DPI	Ovalbumin Days post infection
