# Supplementary material for: Vaccine-Mediated Protection of Mice Against African and Asian Clinical Strains of Cryptococcus neoformans
Source: J Fungi (Basel). 2025 Dec 16;11(12):886. doi: 10.3390/jof11120886 (PMC12733381; doi:10.3390/jof11120886)
Supplement: Supplementary file 1 [file jof-11-00886-s001.zip › jof-3931683-supplementary.pdf]

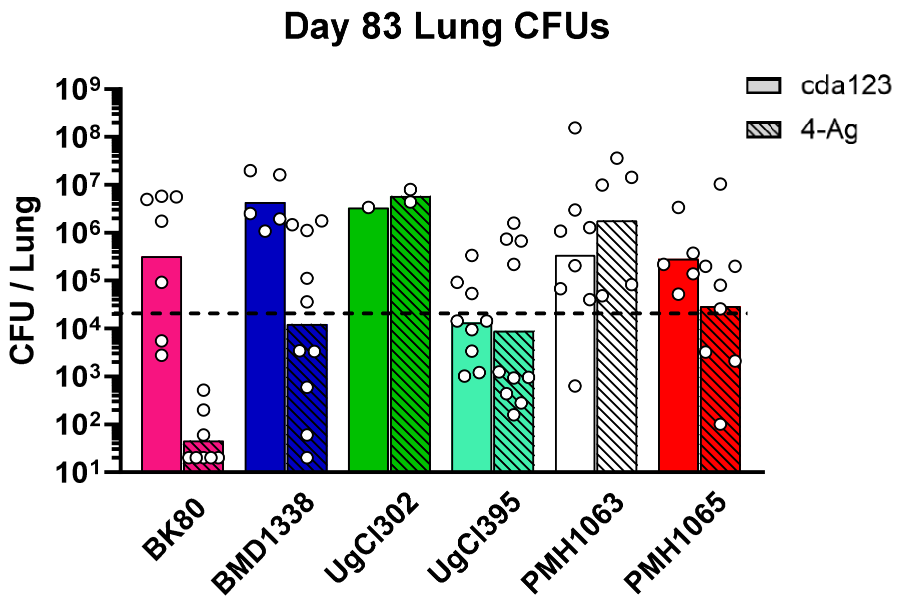

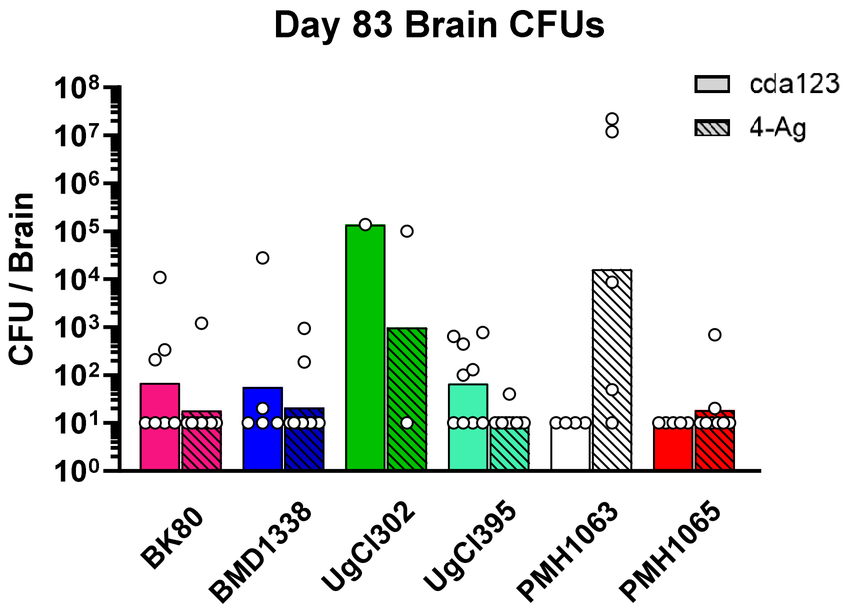

Supplemental Figure S1. Lung and brain fungal burdens of surviving mice following vaccination and infection. Mice received either the live-attenuated cda123 vaccine or the CAF01-adjuvanted Cda1/Cda2 & Cpd1Δ/Blp4 vaccine (4-Ag) as described in the Methods. Mice were challenged orotracheally 2 weeks after the last vaccination with 2 x 10^4 cells of the indicated *C. neoformans* strain. Mice were then monitored for 83 days post infection (DPI) for survival. Survival curves are shown in Figure 2. At the termination of the experiment, surviving mice were euthanized and lung (top) and brain (bottom) CFUs determined. The number of survivors ranged from one to ten depending on the infecting strain and the vaccination. Each

circle represents organ CFUs in an individual mouse. Bars are geometric means. Unvaccinated mice all died prior to day 83 and are not shown in the figure. The level of detection for lungs and brain was 20 CFUs and 10 CFUs, respectively. The horizontal dashed line in the top graph denotes the pulmonary inoculum.

**Strain**	**Vaccine**	
	**Cda123**	**4-Ag**
**BK80**	7/10 M	8/10 F
**BMD1338**	3/5 M, 2/5 F	5/5 M, 5/5 F
**UgCl302**	0/8 F, 1/2 M	2/10 F
**UgCl395**	5/5 F, 4/5 M	10/10 F
**PMH1063**	4/10 F, 4/5 M	4/9 F, 1/1M
**PMH1065**	5/14 F	6/8 F, 2/2 M

Supplemental Table S1. Vaccine Protection as a function of the sex of the mice. The data parse the results shown in Figure 2 according to the sex of the mouse. Results are presented as survivors/total number of mice per group. M, male. F, female. For example, 4/9 F indicates that of 9 vaccinated female mice, 4 were alive at the termination of the study.

**Strain**	**Vaccine**		
	**Unvac**	**Cda123**	**4-Ag**
**BK80**	Closed symbols, all F.<br>Open symbols, all M.	Closed symbols, all F.<br>Open symbols, all M.	Closed symbols, all F.<br>Open symbols, all M.
**UgCl302**	Closed symbols, all F.<br>Open symbols, all M.	All mice were M.	Closed symbols, all F.<br>Open symbols, 3 M, 1 F.
**PMH1063**	All mice were F.	Closed symbols, all M.<br>Open symbols, all F.	Closed symbols, all F.<br>Open symbols, all M.

Supplemental Table S2. Sex of the mice used in Figures 3 and 4. M, male. F, female.
